# Line-Constrained Camera Location Estimation in Multi-Image Stereomatching

**DOI:** 10.3390/s17091939

**Published:** 2017-08-23

**Authors:** Simon Donné, Bart Goossens, Wilfried Philips

**Affiliations:** IPI-UGent-imec, B-9000 Ghent, Belgium; Bart.Goossens@UGent.be (B.G.); Wilfried.Philips@UGent.be (W.P.)

**Keywords:** stereomatching, lightfield images, camera location estimation

## Abstract

Stereomatching is an effective way of acquiring dense depth information from a scene when active measurements are not possible. So-called lightfield methods take a snapshot from many camera locations along a defined trajectory (usually uniformly linear or on a regular grid—we will assume a linear trajectory) and use this information to compute accurate depth estimates. However, they require the locations for each of the snapshots to be known: the disparity of an object between images is related to both the distance of the camera to the object and the distance between the camera positions for both images. Existing solutions use sparse feature matching for camera location estimation. In this paper, we propose a novel method that uses dense correspondences to do the same, leveraging an existing depth estimation framework to also yield the camera locations along the line. We illustrate the effectiveness of the proposed technique for camera location estimation both visually for the rectification of epipolar plane images and quantitatively with its effect on the resulting depth estimation. Our proposed approach yields a valid alternative for sparse techniques, while still being executed in a reasonable time on a graphics card due to its highly parallelizable nature.

## 1. Introduction

Two-image stereomatching has received much attention among passive depth estimation methods, in large part because of the practicality of the set-up. However, multi-image depth estimation has access to more data for estimating the disparity and is hence expected to deliver better results.

### 1.1. The Benefits of Multiple Views

Stereomatching performs depth estimation by detecting objects in two images from a different location. The disparity between these locations is inversely proportional to the distance of the object from the baseline, and directly proportional to the width of the baseline. Obviously, objects that only appear in one of the two views are troublesome: these occlusions are an important problem encountered by two-image stereomatching. Evidence of the expansive existing literature on that topic: it is one of the main discriminating features between the many stereomatching techniques.

Initial methods ignored the problem of occlusions. Many methods, including the seminal work by Horn and Schunk [1], assume that the optical flow field (called the disparity field in stereomatching) is smooth, which means that there are no occlusions [2,3,4,5]. Some methods explicitly detect occlusions (typically based on an intermediate disparity estimate) and handle these regions separately [6,7,8], while others define the problem as an optimization that is more robust to the impact of occlusions through well-chosen cost functions [9,10]. Estimating the disparity field is done by either treating the disparity as a continuous data field [1,2,3,5,6,8,11], or by evaluating a discrete set of hypotheses for each pixel [10,12].

**Figure 1:**
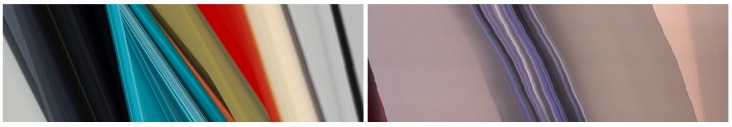
Example epipolar plane images from a camera moving along a dolly in our in-house setup. Both with a constant (left) and variable (right) speed. In the constant case, object edges form straight lines and their depth is related to the angle of this line and the movement speed.

When more than two views of the same scene are available, a more accurate estimation is possible [13,14,15,16]. Similar to these existing methods, we will assume that the camera is moving along a line. The input images are said to be rectified: pixel correspondences between the different images lie on the same scan line. This is either the result of the camera rig (which is what we assume; it is the case for the evaluation datasets in this paper) or can be achieved through rectifying procedures [17,18]. By stacking the same pixel row from multiple camera locations along the baseline, we can generate an epipolar plane image as in Figure 1 [19]. When the cameras are uniformly distributed along the line (the left image, e.g., from plenoptic cameras [20]), depth estimation boils down to estimating the slope of a given pixel’s line. Complete occlusions (i.e., objects only visible in a single image) are much less frequent in such multi-image approaches. A direct disparity estimate is possible for any point visible in at least two of those images.

### 1.2. The Importance of Camera Location Estimation

Current multi-image methods assume that the locations of the cameras are accurately known [14,15,16]. For static set-ups with many cameras (i.e., plenoptic cameras), this is indeed the case. However, for a single moving camera (i.e., on a camera dolly), it is unlikely that the location at any given time is known accurately. We usually do not know these locations accurately because of the non-uniform movement of the camera (see Figure 1). We focus specifically on the scenario of a camera dolly: the camera’s location is physically restricted to a single line, but its velocity is not necessarily well known or even constant.

Methods exist for estimating unconstrained camera positions in video. The method used for estimating camera positions by Kim et al. [15], for example, is the VOODOO camera tracker (VISCODA Gmbh, Hannover, Germany) [21]. It uses sparse image feature correspondences to estimate the camera parameters. Furthermore, it does not explicitly restrict the camera locations to a line, which we know is the case for our camera dolly.

In known rotations, camera resectioning [22,23,24], the location of cameras is estimated under the assumption that their orientations are perfectly known. Our restricted version of the problem, with a constant camera orientation and movement only along the camera *x*-axis, is a more specific version of the known-rotations camera estimation problem, which requires the solution to a large-dimensional homogeneous system.

In this paper, we present a novel approach to estimate the camera locations. The proposed approach fits within the stereomatching mindset: rather than using only a small subset of the input data (sparse image features), we use the entire image as a dense data field for camera location estimation. By exploiting the stereomatching framework, we enjoy the benefit of dense matching (much more measurement points to base the estimation on) without the drawback of having to explicitly take the discreteness of feature points into account. For example, the aperture problem in the optical flow formulation of [1] implies that the cost function contribution of homogeneous areas is nearly constant. For occlusions, the cost function contribution is also relatively flat, but we show that explicitly taking occlusions into account can be done in a straightforward manner. The drawback is, of course, the increased number of computations—we solve this practically by doing all calculations on a graphics card with the aide of the Quasar language [25].

### 1.3. Outline

In [16], we showed how the optical flow approach to dense correspondences [3,5] can be extended to apply to a linear sequence of cameras. We now leverage the same framework to estimate the locations of the cameras involved. Local optima turn out to be an issue, which we mitigate by enforcing monotonicity on the camera locations: the camera only ever moves in one direction. In camera dollies, this is clearly the case, as the camera locations along the movement line follow the image order.

**Figure 2:**
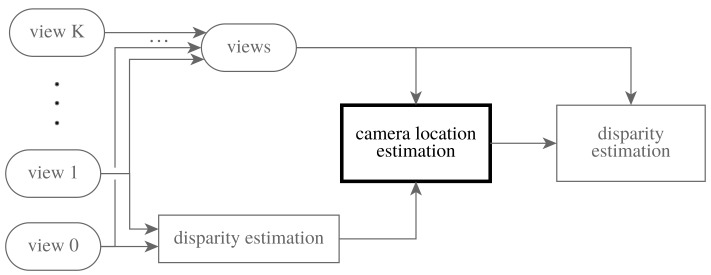
The full disparity estimation work flow. A rough disparity estimate based on two views is used to estimate the camera locations for all views. Afterwards, the camera locations and all input views can be used to estimate a more accurate disparity map. The novel method proposed in this paper concerns the camera location estimation.

The complete work flow for disparity estimation is shown in Figure 2. First, a rough disparity estimate is computed from two views: the central view and the reference view (respectively defining positions 0 and 1 along the line). This initial disparity is used to estimate all of the camera locations $\theta_{k}$, which are required for the full disparity estimation. Using the camera locations $\theta_{k}$, the disparity estimate can be refined using all of the inputs’ views. The novelty of this paper lies in the camera location estimation.

In the (largely hypothetical) scenario that the depth is accurately known, the camera location estimation can be performed directly on the input views and the groundtruth disparity field. In the more likely case that the disparity field is not known in advance, one could envision an iterative process between optimizing the camera locations and refining the estimate of the disparity field—using the refined disparity estimate to improve the location estimates. However, our experiments have shown that there is little to no added value to this workflow, presumably because both optimizations use the same optical flow formulation and hence are already well tuned to each other. When the initial disparity is wrong, this is because of ambiguities in the optical flow formulation (e.g., due to the aperture problem). These ambiguous areas will therefore contribute very little to the cost function of the camera positions (besides adding a nearly constant value).

In Methods, we recap the Multi-image Stereomatching framework from [16] that we adapt and extend in this paper to perform Camera Location Estimation. These are the blocks “disparity estimation” and “camera location estimation” from the diagram in Figure 2, respectively. Results are given, both for known input sequences and for image sequences with non-uniform camera locations. We compare both to the commercially available VOODOO tracker, and the known-rotations solution using the feature points tracked by the VOODOO tracker.

## 2. Methods

### 2.1. Multi-Image Stereomatching

We start with a quick recap of the method in [16], which estimates the disparity fields under the assumption that the camera locations are known. In multi-image stereomatching, we estimate the depth field of the central camera: the disparity of an object between two camera views is inversely proportional to the depth of the object and directly proportional to the distance between the cameras. Formally, we need to estimate the (scalar) disparity field $u_{k}\left(x,y\right)$, which maps each pixel in the image domain Ω in the center view $I_{C}$ to its corresponding pixel in any given view $I_{k}$. As the inputs are rectified, this corresponding pixel lies on the same scanline, i.e., there is only a shift in the horizontal direction. We say that the center view is located at location $\theta_{C}=0$, and therefore $u_{k}\left(x,y\right)$ will be a scaled version of a global disparity field: $u_{k}\left(x,y\right)=\theta_{k}u\left(x,y\right)$. Lighting conditions, vignetting, etc. can cause serious problems for the color constancy assumption. This is why we enforce the correspondence relationship only for a transformed version of the images, $FI_{C}$ and $FI_{k}$:

(1) $$\begin{array}{c} \mathrm{find}u\left(x,y\right)\mathrm{such}\mathrm{that}\forall \left(x,y\right)\in \Omega_{\mathrm{unoccluded}}\mathrm{and}\forall k:\\ FI_{C}\left(x,y\right)=FI_{k}\left(x+\theta_{k}u\left(x,y\right),y\right). \end{array}$$

The image transformation F should preserve the geometry of the input views while mitigating lighting effects; generally speaking, it is a local transform of the pixel values. Good choices are histogram equalisation, a census-transform [26,27], a correlation transform [3], or a conversion to the CIEL${}^{*}a^{*}b^{*}$ colorspace with removal of the luminosity component [7,28].

Finally, the global disparity field u(x,y) is estimated by minimizing a data term $E_{d}$: the sum of squared errors over all views. In order to account for poorly conditioned (i.e., homogeneous) areas or occlusions, a regularisation term $E_{s}\left(u\right)$ is included in the objective function:

(2) $$\min_{u}E_{s}\left(u\right)+E_{d}\left(u,\vec{\theta }\right)=E_{s}\left(u\right)+\sum_{k,x,y}{∥FI_{C}\left(x,y\right)-FI_{k}\left(x+\theta_{k}u\left(x,y\right),y\right)∥}_{2}^{2}.$$

We use the bilateral total variation from [3] as the regularisation term, which has proven to be effective at regularizing the estimate while respecting image borders. For additional details on disparity estimation from known camera locations, please refer to [16].

### 2.2. Camera Location Estimation

The main contribution of this paper is an approach to estimate the camera locations along the baseline based on the data term $E_{d}\left(u,\vec{\theta }\right)$ from Equation (2). We assume that an estimate for the disparity field is known, and we minimize $E_{d}\left(u,\vec{\theta }\right)$ w.r.t. $\vec{\theta }$ rather than *u*.

Without any regularisation or additional constraints on the camera positions, the resulting optimization problem separates into a set of relatively straightforward 1D optimization problems, as the *K* problems become independent. Given an initial estimate $\theta_{k,0}$, we can optimize a small change $\Delta \theta_{k}$ by linearisation around $\theta_{k,0}$ (so that $\theta_{k}=\theta_{k,0}+\Delta \theta_{k}$):

(3) $$\begin{array}{cc} FI_{k}\left(x+\theta_{k}u\left(x,y\right),y\right) & \approx FI_{k}\left(x+\theta_{k,0}u\left(x,y\right),y\right)\\ & +\Delta \theta_{k}.u\left(x,y\right)\partial_{x}FI_{k}\left(x+\theta_{k,0}u\left(x,y\right),y\right). \end{array}$$

We call $FI_{k}\left(x+\theta_{k,0}u\left(x,y\right),y\right)$ the warped version of $FI_{k}x,y$ with the current position estimate; it is calculated by linear interpolation of the values $FI_{k}x,y$ at the locations $\left(x+\theta_{k,0}u\left(x,y\right),y\right)$.

The complete optimization method then consists of a series of such linearisations until convergence.

**Figure 3:**
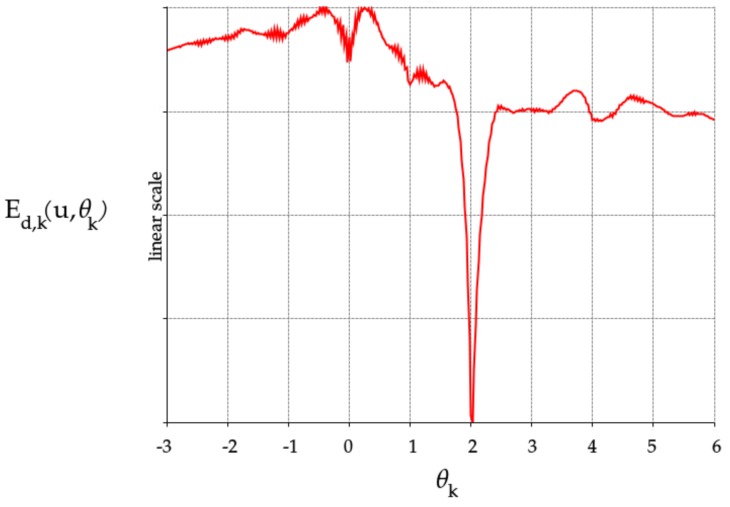
Example cost function of camera location hypotheses. Note the various local minima and the narrow well of attraction around the correct optimum, 2.

Sadly, there turn out to be many local optima for the separate per-camera objective functions (see Figure 3) as well as a relatively small well of attraction near the correct minimum. However, whenever the initial estimates for $\theta_{k,0}$ are somewhat close to their correct values, this approach convergences to the correct optimum.

However, cameras for which the initial estimate is too far away from the correct value are likely to get caught in local minima. In order to not get caught in these local minima, we employ a twofold approach. First of all, we enforce the monotonicity of the camera locations: the order of the cameras along the line is the same as the order of the input images: $\theta_{k}<\theta_{k+1}$. Secondly, we work in a coarse-to-fine manner. In lower resolutions, the global minimum is much wider and local minima are rarer. By enforcing the monotonicity in the lower resolutions, for which the computations are not as expensive, we can mitigate the effect of local minima in the higher resolutions: we can, in the higher resolutions, assume that the estimates are in the vicinity of the correct optimum, and we no longer need the monotonicity constraints.

### 2.3. Monotonicity Constraint

After applying the linearisation, the problem takes the form

(4) $$\min_{\theta_{1},\cdots ,\theta_{K}}\sum_{k}\frac{1}{2}\beta_{k}\Delta \theta_{k}^{2}-\alpha_{k}\Delta \theta_{k},$$

where

(5) $$\begin{array}{cc} \alpha_{k} & =\sum_{(x,y)\in \Omega }\langle FI_{C}\left(x,y\right)-FI_{k}\left(x+\theta_{k,0}u\left(x,y\right),y\right),u\left(x,y\right)\frac{\partial }{\partial x}FI_{k}\left(x+\theta_{k,0}u\left(x,y\right),y\right)\rangle ,\\ \beta_{k} & =\sum_{(x,y)\in \Omega }u{\left(x,y\right)}^{2}{∥\frac{\partial }{\partial x}FI_{k}\left(x+\theta_{k,0}u\left(x,y\right),y\right)∥}_{2}^{2}. \end{array}$$

Note that the values of $\alpha_{k}$ and $\beta_{k}$ depend on the current location estimates $\theta_{k,0}$ and need to be recalculated for each linearization iteration. They are sums over the input image pixels. We take occlusion of the views into account by leveraging the same occlusion detection from [16]; this rsesults in a weight for each pixel, which is 0 whenever the pixel is estimated to be occluded given the current disparity field/location estimate and 1 when it is visible.

As mentioned earlier, without any additional constraints this falls apart in *K* separate problems. Instead, we link all of these problems using monotonicity constraints:

(6) $$\begin{array}{cc} \min_{\theta_{1},\cdots ,\theta_{K}} & \sum_{k}\frac{1}{2}\beta_{k}\Delta \theta_{k}^{2}-\alpha_{k}\Delta \theta_{k}\\ & \mathrm{where}\theta_{k+1}\geq \theta_{k}\;\;\forall k:1\leq k<K. \end{array}$$

Note that this is a quadratic programming problem for which solvers exist. However, we keep in mind that this problem is only an approximation of the actual problem, which is only valid for small $\Delta \theta_{k}$, as it arises from the first order Taylor series expansion in Equation (3). Therefore, accurately solving Equation (6) is of little relevance, and an approximate solution will do. We opt for an iterative approach based on Lagrangian duality. According to the theory of Lagrangian optimisation, this problem is equivalent to minimizing the following Lagrangian w.r.t. $\vec{\theta }$ and $\vec{\lambda }$:

(7) $$\begin{array}{cc} \Lambda (\vec{\theta },\vec{\lambda })= & \sum_{1}^{K}\frac{\beta_{k}}{2}{\left(\theta_{k}-\theta_{k,0}\right)}^{2}-\alpha_{k}\left(\theta_{k}-\theta_{k,0}\right)\\ + & \sum_{1}^{K-1}\lambda_{k}\left(\theta_{k}-\theta_{k+1}\right)\mathrm{with}\lambda_{k}\geq 0, \end{array}$$

and the resulting Lagrangian dual is given by the optimal value of $\theta_{k}$ in function of $\vec{\lambda }$:

(8) $$\begin{array}{c} \theta_{k}^{★}\left(\vec{\lambda }\right)=\theta_{k,0}+\frac{\alpha_{k}+\lambda_{k}-\lambda_{k-1}}{\beta_{k}}. \end{array}$$

Note that this solution $\theta_{k}^{★}\left(\vec{\lambda }\right)$ is similar to the optimum of Equation (4), but corrected with a term to enforce monotonicity: $\left(\lambda_{k}-\lambda_{k-1}\right)/\beta_{k}$. Substituting the expression for $\theta_{k}^{★}\left(\vec{\lambda }\right)$ in Equation (7) results in a quadratic programming problem, which is easier to solve than the quadratic problem from Equation (6) because of the simpler form of the constraints. However, due to the positivity constraints on $\lambda_{k}$, a closed-form solution is not straightforward. We opt instead for iterative Euclidean projection: we calculate the optimal values without the positivity constraints, then project all $\lambda_{k}$ on their feasible set ($R^{+}$). We have found that applying iterative Euclidean projection directly on Equation (6) is not as efficient, and applying it on the dual variables yields better results. The resulting update rules are given by the solution of the system

(9) $$\frac{1}{\beta_{k}}\lambda_{k-1}-\left(\frac{1}{\beta_{k}}+\frac{1}{\beta_{k+1}}\right)\lambda_{k}+\frac{1}{\beta_{k+1}}\lambda_{k+1}=\theta_{k+1,0}-\theta_{k,0}+\frac{\alpha_{k+1}}{\beta_{k+1}}-\frac{\alpha_{k}}{\beta_{k}},\;\forall k\in \left[1,K-1\right].$$

The coefficient matrix of this system is a symmetric tridiagonal matrix, which can be efficiently inverted to solve the system [29,30]. The derivation of the Lagrangian dual in Equation (8) and the system from Equation (9) are given in Appendix A. The Lagrangian optimisation alternates between improving steps in the dual variables $\vec{\lambda }$ using Equation (9) and updates for the locations $\vec{\theta }$ using Equation (8).

### 2.4. Hierarchical Approach

We use a hierarchical approach in order to widen the wells of attraction of the cost function in the initial optimization steps. We subsequently downscale the input images by a factor two until the image size is smaller than 100×100. The camera locations are first estimated in the lowest resolution and iteratively refined on the higher resolution versions of the inputs. The aforementioned monotonicity constraints are only applied on the lower levels; higher levels simply use the closed form solution to Equation (4). This speeds up the optimization as the computations in the lower resolutions are not as expensive. We can assume that for the higher resolutions the estimates are in the vicinity of the correct optimum and no longer need the monotonicity constraints. This strikes a good balance: on the lower levels, where the initial location estimates are likely very poor, we employ the monotonicity constraints to force the location estimates in the right direction. On higher levels, where the initial values $\theta_{k,0}$ are closer to their true value and where calculations are more costly, we adopt the unconstrained estimation from Equation (4).

In traditional optical flow methods, the hierarchical approach is used because the estimation hinges on local first order Taylor series approximations, which means that the update steps should stay smaller. For flow estimation (or stereomatching), the size of the variables scales linearly with the resolution of the input image so that this coarse-to-fine approach helps decrease the step magnitudes. In our proposed location estimation, this is not the reason for the hierarchical approach; however, on lower resolutions, the cost functions are smoother, which results in a better behaviour of iterative optimization techniques.

### 2.5. Overview of the Entire Work Flow

The pseudo code for the full work flow is shown in Algorithm 1. For brevity, all loops over the number of views *K* are omitted, and we simply state the update step for the *k*th entry.

**Algorithm 1:** Overview of the proposed approach  *u*← disparity estimate from two views  $\theta_{k}\leftarrow 0$  $\lambda_{k}\leftarrow 0$  **for**
*t*
**from** T-1 **to** 0 **do**   $u_{t}\leftarrow$ upscale $\frac{u}{f^{t}}$ by factor $f^{t}$ (bilinear)   $FI_{k,t}\leftarrow$ resize $FI_{k}$ by factor $f^{t}$   **for** warp **from** 1 **to** W **do**    $\alpha_{k}\leftarrow \sum_{x,y}u_{t}\left(x,y\right)\langle FI_{C,t}\left(x,y\right)-FI_{k,t}\left(x+\theta_{k}u_{t}\left(x,y\right),y\right),\frac{\partial }{\partial x}FI_{k,t}\left(x+\theta_{k}u_{t}\left(x,y\right),y\right)\rangle$    $\beta_{k}\leftarrow \sum_{x,y}u_{t}{\left(x,y\right)}^{2}{∥\frac{\partial }{\partial x}FI_{k,t}\left(x+\theta_{k}u_{t}\left(x,y\right),y\right)∥}^{2}$    **for**
*ℓ*
**from** 1 **to** L **do**     $\lambda_{k}\leftarrow$ the solution of the system Equation (9) (see Appendix A)    **end for**    $\theta_{k}\leftarrow \theta_{k,0}+\frac{\alpha_{k}+\lambda_{k}-\lambda_{k-1}}{\beta_{k}}$   **end for**  **end for**  *u*← disparity estimate from all views as described in [16].

First, we estimate the disparity for the center view from the center view and the reference view (fixed at, respectively, locations 0 and 1). Then, for each level *t* of the pyramid, we perform *W* iterations of the linearisation around the current estimates of $\theta_{k}$. Each such iteration is called a warp because it entails warping the images with the current estimate of the disparity field $\theta_{k}u\left(x,y\right)$: $FI_{k,t}\left(x+\theta_{k}u_{t}\left(x,y\right),y\right)$. Per warp, we require the computation of the new values for $\vec{\lambda }$ and those of $\vec{\theta }$. The values for $\vec{\lambda }$ follow from *L* iterations of block coordinate descent, while the optimal values for $\vec{\theta }$ are given in closed form by Equation (8).

Finally, the estimated camera locations are used in a multi-image stereomatching algorithm to arrive at a disparity estimate of the scene.

The parameters used for the reported results are:

- T=5 levels in the pyramid, with a scaling factor of f=2;
- W=10 warps per level;
- L=10 block-coordinate descent iterations per warp for optimizing the dual variables.

The bottleneck of the algorithm is by far the calculation of $\alpha_{k}$ and $\beta_{k}$ in each warp because of the summation over all of the image pixels. The method was implemented and executed on the Graphic Processing Unit (GPU) using the Quasar programming language [25], speeding up these calculations drastically compared to a Central Processing Unit (CPU) implementation.

## 3. Results

Our evaluation of the proposed method is fourfold. First of all, we take a look at the monotonicity constraint and illustrate its effectiveness at estimating camera positions far from the reference camera position (the one we define to be at location 0). Secondly, we quantify the accuracy of the proposed method in terms of estimated camera positions. We compare on both existing datasets from Kim et al. [15] and new datasets captured in the context of this paper. In the third subsection, we illustrate the method’s effectiveness at estimating the camera parameters in a practical setting, plus how—and why—it can cope with typical stereomatching artifacts and errors. Finally, we show visually how the proposed method transforms non-uniformly sampled epipolar plane images into rectified versions consisting only of straight lines.

### 3.1. Monotonicity Constraint

In our first experiment, we estimate the camera positions twice: once with and once without the monotonicity constraints on the locations. To this end, we use some datasets from [15]: the *statue* and *couch* data sets. Kim et al. provide a large set of rectified views for both of these sets, as well as estimated disparity maps.

**Figure 4:**
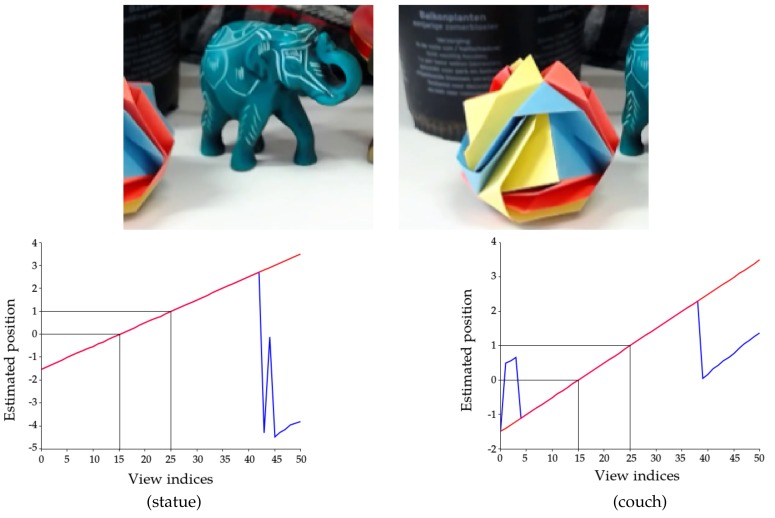
Example input to the algorithm. Top: cropping the images results in more occlusions and a more challenging location estimation. Bottom: the graphs show the unconstrained Solution (4) (in blue) and the constrained Solution (6) (in red) using the high quality disparity maps from [15] as input, for the cropped statue (left) and cropped couch (right) datasets.

For a more challenging scenario, we use details of these images. This means that, for large displacements of the camera, the scene will change noticeably. We do this by extracting the 640×480 patches with the top-left corner at respectively (900,800) for statue and (800,2000) for couch. Cropping results in relatively larger homogeneous areas and more occlusions. This is illustrated in Figure 4.

We perform the camera location estimation using the input disparity maps estimated in [15]. When the unconstrained location estimate is correct, we see in Figure 4 that the constrained estimate simply adopts the same values. When the unconstrained optimization converges poorly, the monotonicity constraints guide the camera locations and we arrive at a correct estimate (see the graphs in Figure 4).

### 3.2. Accuracy of the Proposed Method

In order to quantify the accuracy of the proposed method, we compare its performance with that of the VOODOO camera tracker. We do this both on the Disney datasets and three additional datasets captured in the context of this publication: map, office and collection. These datasets were recorded with a camera mounted on a rail. The movement speed of the cameras was hence not uniform. The rail was annotated with a ruler, and we halted the camera for a while after every 5 mm (map and collection) or 1 cm (office). We automatically extracted the frames with the least inter-frame changes near those locations and visually inspected the videos for correctness.

For the baseline, we evaluate three distinct methods. The first one, VOODOO projected, is the one implemented by the VOODOO tracker. It tracks feature points through the sequence, and then performs unconstrained structure-from-motion estimation. The resulting camera position estimates are regressed onto a single line. The second one, VOODOO constrained, uses the same tracked features (already filtered from outliers and with correspondence information), and passes them to a known-rotations problem solver. As we know that the orientation is fixed and that the cameras only move along their own *x*-axis, we only need estimate a single value for each camera and a 3D location for each distinct point. The result is a linear homogeneous system that is solved by a singular value decomposition. Additionally, we also compare to our proposed method with the ground truth disparity as input. Finally, a note on the location reference systems is provided. While our proposed approach assumes the central camera to be at position 0, and an arbitrarily chosen reference camera at position 1, this is not the case for the VOODOO methods. They have an unknown reference system for their camera positions, which we do not know and which makes quantitative validation hard. For this reason, both of them are subjected to an additional optimization over the origin and the step size of this axis (see the Appendix for more details). The displayed values are those for the best-case reference system.

**Figure 5:**
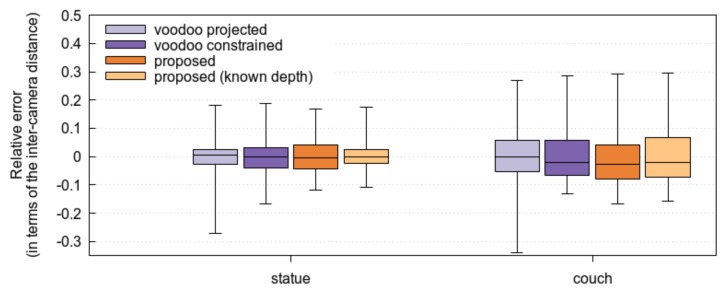
Comparison of the proposed method and the VOODOO tracker, on the cropped version of the Disney datasets couch and statue. The (proposed, 1) entry denotes the estimation using the disparity maps provided by Kim et al., while (proposed, 2) denotes the estimation with disparity maps estimated as in [16]. The box plot shows the average camera location error expressed as a fraction of the inter-camera distance (which is the same for all adjacent cameras), as well as the quantiles.

As is visible in Figure 5, the proposed method matches the VOODOO tracker whether it starts from disparity maps estimated by Kim et al., or disparity maps estimated by our earlier method [16].

**Figure 6:**
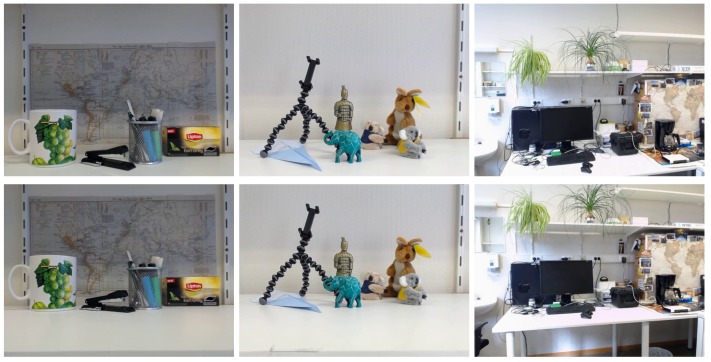
The extreme camera positions for three datasets captured in our lab (from left to right: the map, collection and office datasets). The datasets were recorded using a rail-mounted camera (using a crank and chain system). For the map and collection datasets, the distance to the scene is roughly 20–30 cm and the inter-camera distance is 5 mm between all views. For the office dataset, the distance to the scene is roughly 2–5 m and the inter-camera distance is 1 cm between all views. As our set-up has the camera moving vertically, the algorithms described here are applied on the transposed images, and all images are transposed again for visualization.

**Figure 7:**
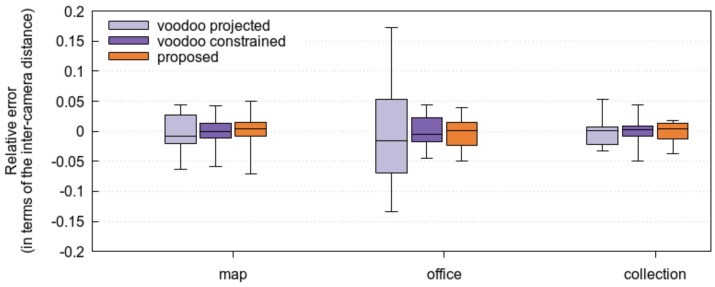
Comparison of the proposed method and the VOODOO tracker, on the three datasets recorded in our lab. The used disparity maps were all estimated as in [16]. The box plot shows the average camera location error expressed as a fraction of the inter-camera distance (between the center and the reference camera), as well as the quantiles.

Clearly, the case of known disparity maps is an atypical case and we need to evaluate the method when it uses estimated disparity maps as well. For our own test scenarios (see Figure 6), no high-quality disparity maps are available. Therefore, we first perform the disparity estimation on one pair of images, and then use this estimate of the disparity for the location estimation. We regress VOODOO’s locations estimates to a single line and project all camera locations on that line to arrive at a set of linear locations estimates for the cameras. In Figure 7, it is visible that here, too, our proposed method fares better than the VOODOO tracker.

### 3.3. Estimation in a Practical Setting

**Figure 8:**
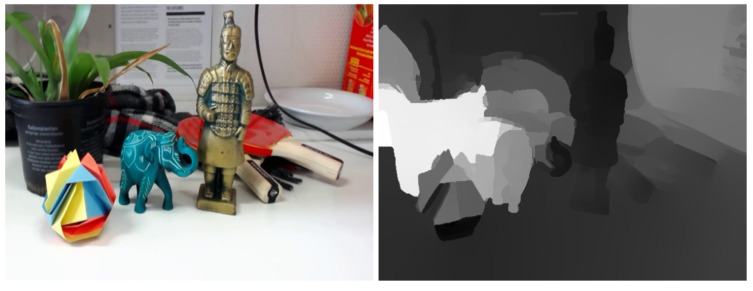
Example frame and initial two-view depth estimate for a manually recorded sequence. The initial image as well as its rectified version (using this depth estimate as input) are visible in Figure 9a.

**Figure 9:**
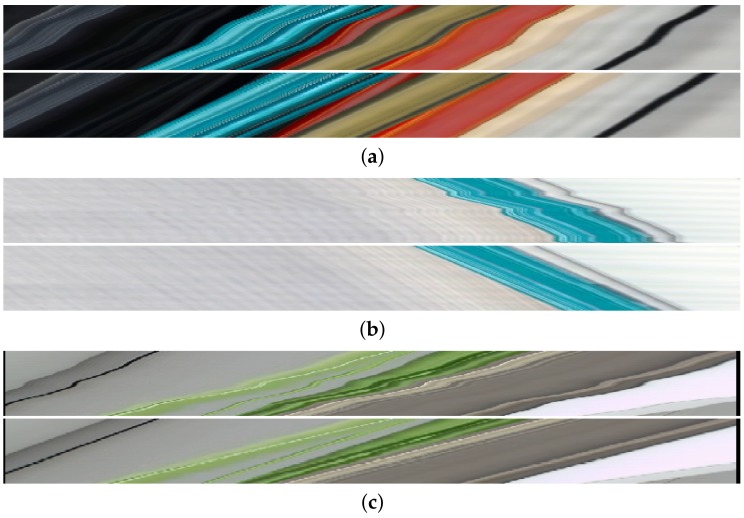
Input epipolar plane images and their rectified versions after location estimation. In a rectified epipolar plane image (of a static scene), all lines are straight. When the camera speed is not constant, this is not the case and we can use the estimated camera locations to resample the images. We show the inputs and outputs of this process for three different captures. It is visible that, indeed, the non-uniform movement is correctly rectified and the (lower) rectified images are comprised of straight lines. (**a**) Original and rectified epipolar plane image for the sequence of Figure 8; (**b**) Original and rectified epipolar plane image for a sequence with reversed movement. Here we simply flip the images horizontally and invert the sign of the estimated positions; (**c**) Due to the global nature of the position estimation, thin lines such as the black wire on the left or the thin green leaf in the middle are rectified just as easily as larger objects.

In order to test the proposed approach in a practical set-up, we have used a rail-mounted camera (propelled manually) to capture a static scene. The camera’s motion is linear and perpendicular to its look direction, such that the prerequisites of the method are fulfilled. Consider an example input frame and its two-view depth estimate from stereomatching as given in Figure 8. This is the input depth map that was used to estimate the locations of the cameras and rectify the epipolar plane images as given in Figure 9 (i.e., resample them by simulating uniformly distributed camera positions). It is illustrated that, while the entire left portion of the image suffers from a poor disparity estimate, the correct right half still results in correctly estimated camera locations. Additionally, the monotonicity constraints force the location estimates to enter the neighbourhood of the local minima. We note that, while the disparity estimate appears completely off, it was estimated using the method on which our location estimation is based [16]. That is to say, the areas where the estimate is off are those where stereomatching itself is ill-defined (i.e., occlusions or homogeneous areas). In essence, this means that, for that area, it is hard to discriminate between various depth estimates and, by extension, those areas will hold little sway over the camera position estimation. Hence, our approach is robust to such errors in the input map.

**Figure 10:**
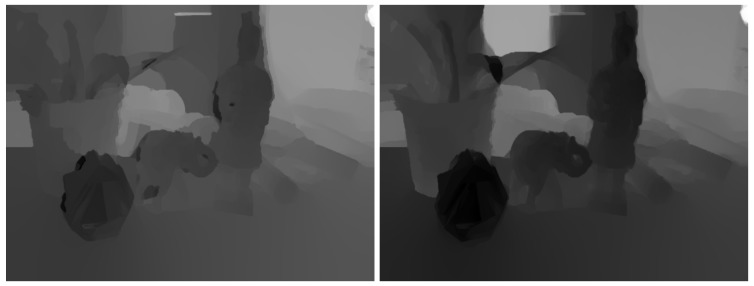
The multiview estimation for the scene from Figure 8. Both for wrong locations (assumed uniform, left) and after our proposed location estimation (right).

We also show the impact of a correct location estimation. Figure 10a shows the estimated disparity for the manually recorded sequence, using all input views. The left image shows the estimated disparity field based on a faulty location hypothesis: uniform camera speed. The right image shows the disparity estimate when using our proposed approach to estimate the camera locations.

One can see that the estimation exploiting multiple views (with the correct camera locations) is able to avoid the issues in the left half of the input frame that were present in the two-view scenario in Figure 8. Moreover, one can also see that Figure 10a contains errors that are not present in Figure 10b, e.g., the depth of the elephant, or near the edges of the statue. Correct estimation of the camera positions is paramount for the depth estimation.

### 3.4. Rectifying the Epipolar Plane Images

Figure 9 shows the images before and after rectification. Rectification consists of the vertical resampling of the epipolar plane image, given the estimated camera locations, which are used as the actual row locations—we resample to uniform row locations: a perfectly rectified epipolar plane image contains only straight lines and constant areas. In effect, we linearily resample to virtual camera locations uniformly over the camera movement line (between both extreme camera positions). These rectified versions can be used by methods that assume uniform camera movement, such as the estimation of the slopes of the lines by [15].

## 4. Conclusions

In this paper, we propose a novel technique to estimate camera locations in a multi-view stereomatching set-up. The technique is based on the optical flow formulation of the stereomatching problem, which allows us to express the location estimation as the minimization of a second-order polynomial. After experimental motivation that local minima can be an issue in this optimization problem, we introduce monotonicity constraints to mitigate them.

Estimation of the camera locations is done using a primal-dual approach based on the theory of Lagrangian duality. We perform estimation in a coarse-to-fine manner in order to only have the cost of the expensive primal-dual optimization in the lower resolutions. In higher resolutions, we can assume that we are already in the vicinity of the global optimum, and we do not require monotonicity constraints. The proposed method uses an input disparity estimate for the scene, but it is motivated and illustrated to be robust against poor input disparity estimates because of the shared optical flow formulation: ambiguous areas for stereomatching are also ambiguous for the camera position estimation, and hence influence the final estimate only a very little amount. Finally, we illustrate the impact and importance of the accuracy of the camera location estimates, by performing disparity estimation on a manually recorded scene, both with poor camera location guesses and with the estimated camera locations. Quantitatively, the methods delivers results that match and improve those from existing sparse feature-based trackers.

## Appendix A. Deriving the Update Rules

We now derive the Lagrangian dual from Equation (8) and the update rule for the dual variables $\vec{\lambda }$. Recall that the optimization problem is given by:

(A1) $$\min_{\vec{\theta }}\sum_{1}^{K}\frac{1}{2}\beta_{k}{\left(\theta_{k}-\theta_{k,0}\right)}^{2}-\alpha_{k}\left(\theta_{k}-\theta_{k,0}\right),\mathrm{where}\theta_{k}\leq \theta_{k+1}\;\forall k\in \left[1,K-1\right].$$

The corresponding Lagrangian dual problem is given by

(A2) $$\max_{\vec{\lambda }}\min_{\vec{\theta }}\Lambda \left(\vec{\theta },\vec{\lambda }\right)=\sum_{1}^{K}\frac{1}{2}\beta_{k}{\left(\theta_{k}-\theta_{k,0}\right)}^{2}-\alpha_{k}\left(\theta_{k}-\theta_{k,0}\right)+\lambda_{k}\left(\theta_{k}-\theta_{k+1}\right),$$

where $\lambda_{k}\geq 0,\;\forall k\in \left[1,K-1\right]$ and $\lambda_{K}=0$. Deriving with respect to $\theta_{k}$ yields

(A3) $$\partial_{\theta_{k}}\Lambda \left(\vec{\theta },\vec{\lambda }\right)=\beta_{k}\left(\theta_{k}-\theta_{k,0}\right)-\alpha_{k}+\lambda_{k}-\lambda_{k-1}.$$

Solving the system $\partial_{\vec{\theta }}\Lambda \left(\vec{\theta },\vec{\lambda }\right)=\vec{0}$ results in the Lagrangian dual

(A4) $$\theta_{k}^{★}\left(\vec{\lambda }\right)=\theta_{k,0}+\frac{\alpha_{k}-\lambda_{k}+\lambda_{k-1}}{\beta_{k}}.$$

We are thus adjusting the regularization-free estimation ($\theta_{k,0}+\alpha_{k}/\beta_{k}$) with the regularization term $\left(\lambda_{k-1}-\lambda_{k}\right)/\beta_{k}$. Plugging this Lagrangian dual back into the Lagrangian function yields the dual function $\Lambda \left({\vec{\theta }}^{★}\left(\vec{\lambda }\right),\vec{\lambda }\right)$. This is a quadratic programming problem; it lacks a closed form solution because of the positivity constraints on $\lambda_{k}$. Because of the low complexity of the dual function, block coordinate descent is an adequate optimization technique. Omitting the dependency of ${\vec{\theta }}^{★}$ on $\vec{\lambda }$, the dual function’s gradient is given by

(A5) $$\partial_{\lambda_{j}}\Lambda \left({\vec{\theta }}^{★}\left(\vec{\lambda }\right),\vec{\lambda }\right)=\sum_{1}^{K}\beta_{k}\left(\theta_{k}^{★}-\theta_{k,0}\right)\partial_{\lambda_{j}}\theta_{k}^{★}-\alpha_{k}\partial_{\lambda_{j}}\theta_{k}^{★}+\lambda_{k}\left(\partial_{\lambda_{j}}\theta_{k}^{★}-\partial_{\lambda_{j}}\theta_{k+1}^{★}\right)+\left(\theta_{k}^{★}-\theta_{k+1}^{★}\right)\partial_{\lambda_{j}}\lambda_{k}.$$

This simplifies to

(A6) $$\partial_{\lambda_{j}}\Lambda \left({\vec{\theta }}^{★}\left(\vec{\lambda }\right),\vec{\lambda }\right)=\theta_{j,0}-\theta_{j+1,0}+\frac{\alpha_{j}-\lambda_{j}+\lambda_{j-1}}{\beta_{j}}+\frac{-\alpha_{j+1}+\lambda_{j+1}-\lambda_{j}}{\beta_{j+1}}.$$

By equating all partial derivatives to zero, we end up with the following system:

(A7) $$\left[\begin{array}{ccccccc} & & & \cdots \\ \cdots & 0 & \frac{1}{\beta_{k}} & -\left(\frac{1}{\beta_{k}}+\frac{1}{\beta_{k+1}}\right) & \frac{1}{\beta_{k+1}} & 0 & \cdots \\ & & & \cdots \end{array}\right]\left[\begin{array}{c} \vdots \\ \lambda_{k-1}\\ \lambda_{k}\\ \lambda_{k+1}\\ \vdots \end{array}\right]=\left[\begin{array}{c} \vdots \\ \theta_{k+1,0}-\theta_{k,0}+\frac{\alpha_{k+1}}{\beta_{k+1}}-\frac{\alpha_{k}}{\beta_{k}}\\ \vdots \end{array}\right].$$

Here, the coefficient matrix is a symmetric tridiagonal matrix that can be efficiently inverted [29,30].

## Appendix B. Optimizing the Location Reference System for the Trackers

Our proposed approach assumes the central camera *C* to be at position 0 and an arbitrarily chosen reference camera *R* at position 1. Given this reference system, we can express the mean squared error of the camera positions (in terms of the inter-camera distance) as:

(A8) $${\mathrm{MSE}}_{\mathrm{proposed}}\left(\vec{\theta }\right)=\sum_{k}{\left(\theta_{k}-\frac{k-C}{R-C}\right)}^{2}.$$

However, this is not the case for the VOODOO methods. They have an unknown reference system for their camera positions, which we do not know and which makes quantitative validation hard. Therefore, we optimize over the offset and scale of that reference system in order to quantify the mean squared error (MSE): for these methods:

(A9) $${\mathrm{MSE}}_{\mathrm{VOODOO}}\left(\vec{\theta }\right)=\min_{\mathrm{offset},\mathrm{scale}}\sum_{k}{\left(\theta_{k}-\frac{k-\mathrm{offset}}{\mathrm{scale}}\right)}^{2}.$$
